# *Tango7* regulates cortical activity of caspases during *reaper*-triggered changes in tissue elasticity

**DOI:** 10.1038/s41467-017-00693-3

**Published:** 2017-09-19

**Authors:** Yunsik Kang, Sarah D. Neuman, Arash Bashirullah

**Affiliations:** 1 Division of Pharmaceutical Sciences, University of Wisconsin-Madison, 777 Highland Avenue, Madison, WI 53705-2222 USA; 2 Laboratory of Genetics Graduate Program, University of Wisconsin-Madison, Madison, WI 53705 USA; 3 Cellular and Molecular Biology Graduate Program, University of Wisconsin-Madison, Madison, WI 53705 USA; 4 Present Address: Vollum Institute, Oregon Health & Sciences University, Portland, OR 97239-3098 USA

## Abstract

Caspases perform critical functions in both living and dying cells; however, how caspases perform physiological functions without killing the cell remains unclear. Here we identify a novel physiological function of caspases at the cortex of *Drosophila* salivary glands. In living glands, activation of the initiator caspase *dronc* triggers cortical F-actin dismantling, enabling the glands to stretch as they accumulate secreted products in the lumen. We demonstrate that *tango7*, not the canonical Apaf-1-adaptor *dark*, regulates *dronc* activity at the cortex; in contrast, *dark* is required for cytoplasmic activity of *dronc* during salivary gland death. Therefore, *tango7* and *dark* define distinct subcellular domains of caspase activity. Furthermore, *tango7*-dependent cortical *dronc* activity is initiated by a sublethal pulse of the inhibitor of apoptosis protein (IAP) antagonist *reaper*. Our results support a model in which biological outcomes of caspase activation are regulated by differential amplification of IAP antagonists, unique caspase adaptor proteins, and mutually exclusive subcellular domains of caspase activity.

## Abstract (web summary)

Caspases are known for their role in cell death, but they can also participate in other physiological functions without killing the cells. Here the authors show that unique caspase adaptor proteins can regulate caspase activity within mutually-exclusive and independently regulated subcellular domains.

## Introduction

Caspases are cysteine-aspartic proteases best known for their roles in initiating cellular demolition during apoptosis^1, 2^. These proteins are maintained within the cell as inactive zymogens. However, once activated, a caspase cascade is released, resulting in critical consequences for the cell. Initiator caspases sit at the top of this cascade. These proteins exist in the cell as inactive monomers, but upon receipt of an activating stimulus, they form active dimers, mediated by adaptor proteins^3^. The best characterized activation platform, which is required during apoptosis, is the apoptosome^4, 5^. The apoptosome consists of the initiator caspase, caspase-9 (*dronc* in *Drosophila*), and the adaptor protein Apaf-1 (*dark* in *Drosophila*). In mammals, release of cytochrome c activates the apoptosome, allowing caspase-9 to cleave a second class of caspases, the effector caspases. The effector caspases exist in the cell as inactive dimers, and require cleavage by initiator caspases for activation^6, 7^. The major effector caspases in *Drosophila* are *drice* and *dcp-1*. Activated effector caspases then cleave critical cellular targets as well as other effector caspases, generating a cascade of caspase activity that eventually results in cellular demolition. This self-perpetuating cascade of caspase activation led to the widespread notion that caspase activation represents a “point of no return” in the life of a cell. However, over the last decade, there have been many examples of caspases playing non-lethal, or “non-apoptotic,” roles in cells that do not die.

The list of non-lethal, non-apoptotic roles of caspases has been steadily growing^8–13^. For example, the initiator caspase, caspase-9, and the effector caspase, caspase-3, have been shown to mediate axon pruning during local deprivation of NGF^14^. Additionally, mouse hair follicles and the surrounding cells require non-apoptotic functions of the effector caspase, caspase-7, for proper development^15^. In *Drosophila*, the initiator caspase *dronc* plays a critical role in dendrite pruning of the sensory neurons of the peripheral nervous system^16, 17^, and also plays an important role in sperm individualization^18–20^. Although many non-apoptotic functions of caspases have been identified, how caspases function without executing the cell has remained a mystery. Unfortunately, these lethal and non-lethal outcomes of caspase activation have been studied in different cell types, making mechanistic comparisons very difficult.

We have found that the *Drosophila* larval salivary glands provide an ideal model to study developmentally regulated non-lethal and lethal functions of caspases in a single cell type. Here we examine two distinct caspase activation events during salivary gland development: one resulting in a non-apoptotic, non-lethal outcome and the second resulting in a lethal outcome. We find that these two events are both regulated by the steroid hormone ecdysone; however, differential signaling mechanisms selectively amplify the activating signal, IAP antagonist expression, to generate a lethal outcome instead of a non-lethal response. Moreover, we also demonstrate that caspases can be activated in mutually exclusive subcellular domains to accomplish different biological functions, and the use of different adaptor proteins mediates this mutually exclusive activation. Finally, our results highlight a novel, non-lethal function for caspases in the control tissue elasticity during exocrine secretion events. Altogether, we provide a new model for how caspases can be activated and perform cellular functions without triggering cell death during development.

## Results

### A regulated sublethal pulse of *rpr* in salivary glands

**Fig. 1:**
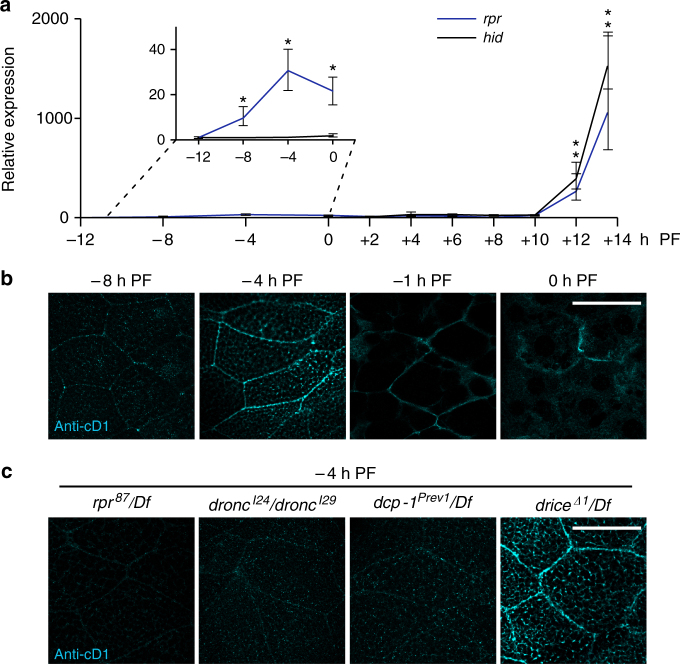
A low amplitude pulse of *reaper* (*rpr*) triggers caspase activation at the cortex of salivary glands. **a** qPCR analysis of *rpr* (*blue line*) and *head involution defective* (*hid*) (*black line*) expression in salivary glands at the onset of metamorphosis. Both *rpr* and *hid* are induced >1000-fold at the start of pupal development, while only *rpr* is induced (~ 30-fold) at the end of larval development. *y-*axis shows relative expression; *x-*axis shows developmental stage in hours relative to the onset of metamorphosis. Expression levels shown relative to the lowest point for each gene and normalized to the reference gene *rp49*. Three biological samples analyzed for each stage; *error bars* represent standard error determined by REST analysis (see “Methods”); asterisks indicate *p-*value < 0.05 calculated by REST analysis. **b** Timecourse of cleaved-Dcp1 (anti-cD1) antibody staining in salivary glands at the end of larval development. Anti-cD1, in *cyan*, does not exhibit specific staining at −8 h PF, but stains strongly at the cortex at −4 h PF. Cortical staining of cD1 persists through −1 h PF, but diminishes at 0 h PF. **c** Regulation of anti-cD1 cortical staining in salivary glands at −4 h PF. Cortical cD1 staining is absent in *rpr*, *dronc*, and *dcp-1* mutant salivary glands, but present in *drice* mutant salivary glands. *Scale bars* represent 100 µm. PF, puparium formation, Df, deficiency

In *Drosophila*, caspase activation hinges on transcriptional induction of IAP antagonists^21^, proteins that remove inhibitor of apoptosis proteins (IAPs) and initiate a caspase cascade. The primary IAP antagonists in *Drosophila* are *reaper* (*rpr*) and *head involution defective* (*hid*), and these proteins play a critical role in the programmed cell death of the larval salivary glands during metamorphosis^22, 23^. Our gene expression studies in the larval salivary glands at the onset of metamorphosis revealed a 1000-fold induction of *hid* at the start of pupal development (Fig. 1a). In contrast, we observed two distinct pulses of *rpr* expression: a 30-fold induction at the end of larval development, and a 1000-fold induction at the start of pupal development (Fig. 1a). The late, large pulse of *rpr* and *hid* has previously been characterized as part of the larval salivary gland cell death response;^22, 23^ however, the early, small pulse of *rpr* has not been described before. We wanted to confirm that this small *rpr* pulse was biologically relevant, so we first tested if the pulse was developmentally regulated. The large, lethal pulse of IAP antagonists is induced by the prepupal pulse of the steroid hormone 20-hydroxyecdysone (henceforth called ecdysone)^23^. Another ecdysone pulse occurs at the end of larval development^24^, and peak steroid hormone levels coincide with the timing of the small pulse of *rpr* expression. We therefore tested if this small *rpr* pulse was regulated by ecdysone signaling. We found that tissue-specific expression of a dominant negative form of the ecdysone receptor (*EcR* ^*F645A*^) abolished *rpr* expression at the end of larval development (Supplementary Fig. 1a), indicating that this small *rpr* pulse is developmentally regulated by the late larval pulse of ecdysone.

Although ecdysone signaling initiates induction of both the small and large *rpr* pulses, the mechanisms mediating the difference in magnitude between these pulses were unclear. We tested if this expression difference was regulated by different downstream targets of ecdysone. Several transcription factors, including *BR-C*, *E74A*, and *Med24*, are required for salivary gland cell death, and these transcription factors are themselves induced by ecdysone^23, 25, 26^. We found that *BR-C*, *E74A*, and *Med24* mutant salivary glands had reduced expression of *rpr* at the late, lethal pulse (Supplementary Fig. 1b). In *BR-C* mutant salivary glands (*rbp* ^5^), this resulted in *rpr* expression levels that resembled the magnitude of the early, small larval pulse. Interestingly, these same three mutants did not affect *rpr* expression at the small, early pulse (Supplementary Fig. 1a). Taken together, these results indicate that downstream targets of ecdysone, like *BR-C*, *E74A*, and *Med24*, differentially amplify *rpr* expression during the death response, making it possible to generate both sublethal and lethal pulses of the IAP antagonist. Furthermore, the early, sublethal pulse of *rpr* was induced in a tissue-specific manner. Although all tissues tested responded to ecdysone by inducing expression of the primary target gene *E74A*, only the salivary glands and midgut induced *rpr* expression, while the wing discs and central nervous system did not (Supplementary Fig. 2). These results suggest that lethal vs. sublethal pulses of *rpr* are developmentally controlled, raising the intriguing possibility that differential amplification and tissue-specific expression of IAP antagonists may play a role in determining apoptotic vs. non-apoptotic outcomes of caspase activation.

### Sublethal *rpr* pulse initiates cortical caspase activation

To determine if the sublethal pulse of *rpr* in salivary glands had a function, we first examined if caspases were activated at this stage. We have previously shown that staining glands at this stage with antibodies directed to cleaved caspase-3 (anti-cC3) does not show any signs of caspase activation^27^. Staining with antibodies directed to cleaved-Dcp-1 (anti-cD1), however, showed staining primarily at the cell cortex, with the intensity of staining peaking at −4 h PF (4 h before puparium formation) (Fig. 1b). This Dcp-1 activation profile coincides with the peak of *rpr* expression, suggesting that the two events may be related. Indeed, in *rpr* mutant glands, anti-cD1 staining was disrupted (Fig. 1c). Furthermore, anti-cD1 staining required the initiator caspase *dronc* and the effector caspase *dcp-1*, but not the effector caspase *drice* (Fig. 1c). Importantly, these results indicate that the small, sublethal pulse of *rpr* initiates activation of specific caspases at the cortex of developing larval salivary glands.

### Caspases dismantle cortical F-actin in living glands

**Fig. 2:**
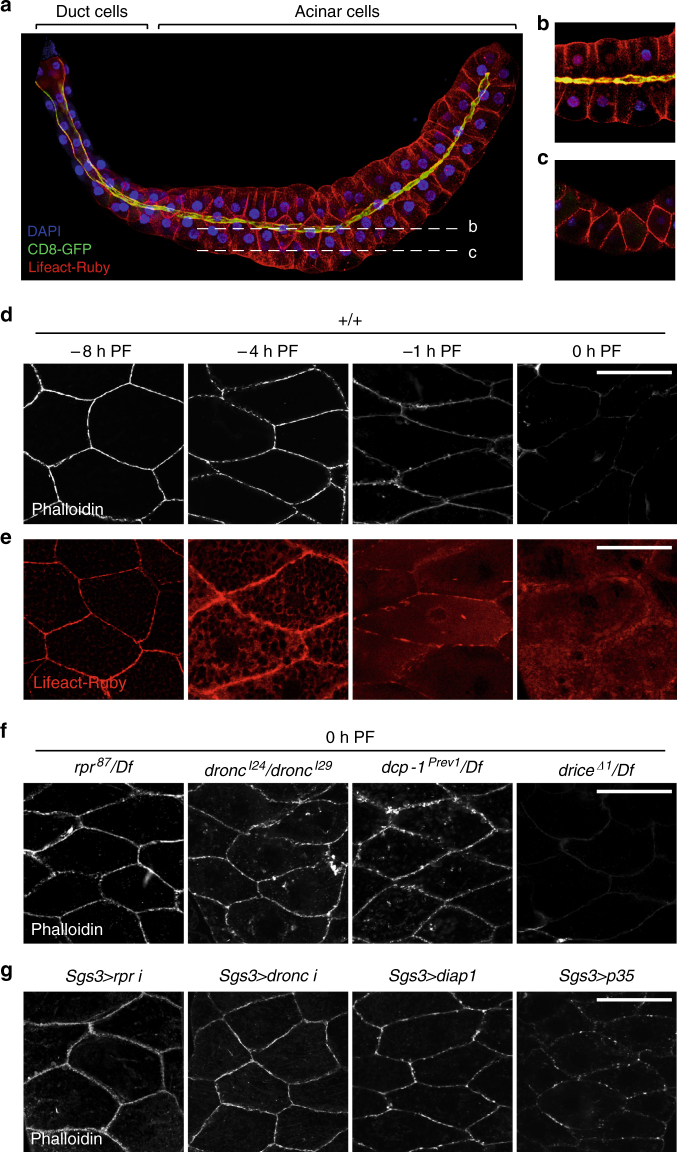
A *rpr*-triggered caspase cascade dismantles the cortical F-actin cytoskeleton in living salivary glands. **a** Maximal intensity projection of a whole salivary gland at −8 h PF expressing the actin-binding peptide Lifeact-Ruby in *red* and the membrane marker CD8-GFP in *green*, both under control of the salivary gland driver *fkh-GAL4*. CD8-GFP is enriched at the apical membrane (proximity of Lifeact-Ruby and CD8-GFP at the apical membrane appears *yellow*); nuclei stained by DAPI in *blue*. **b**, **c** Confocal slices through the salivary glands. **b** A medial slice shows an apical–basal view of acinar cells, with the lumen in the middle. **c** A lateral slice shows a “coronal” view, highlighting the cortical F-actin cytoskeleton. **d**, **e** Cortical F-actin breaks down at the end of larval development. **d** Phalloidin staining (*white*) in salivary glands at −8 h PF reveals tight cortical bundles of F-actin; this structure begins to disintegrate at −4 h PF and −1 h PF, with no phalloidin staining visible by 0 h PF. **e** Lifeact-Ruby in salivary glands confirms cortical F-actin breakdown. Lifeact-Ruby reveals that most of the actin in the cell is present at the cortex at −8 h PF. Breakdown of F-actin is reflected by a gradual redistribution of Lifeact-Ruby signal from the cortex to the cytoplasm at −4 h PF and −1 h PF, with only cytoplasmic signal visible at 0 h PF. **f** F-actin dismantling requires caspase activity. F-actin breakdown is blocked in *rpr*, *dronc*, and *dcp-1* mutant salivary glands, but not in *drice* mutant salivary glands. **g** Caspases regulate F-actin breakdown cell-autonomously. Salivary gland-specific (using *Sgs3-GAL4*) overexpression of *rpr-RNAi*, *dronc-RNAi*, or of the caspase inhibitors *diap1* or *p35* inhibits F-actin breakdown. *Scale bars* represent 100 µm. Df, deficiency, PF, puparium formation

To identify the consequence of caspase activation at the cortex of the larval salivary glands, we tested several vital dyes and antibodies known to stain the cell cortex. Only phalloidin, a vital dye that binds to filamentous actin (F-actin), showed a dramatic difference in staining between −8 h PF and 0 h PF glands. The cortex of most living cells features a meshwork of F-actin bundles that confers shape and structural stability to the cell. Accordingly, we observed a meshwork of cortical F-actin framing every cell in the larval salivary glands (Fig. 2a). The acinar cells of the salivary gland form a radially symmetric polarized epithelium; the apical membrane, visualized by expression of a membrane-targeted GFP (CD8-GFP) (Fig. 2a, b), outlines the lumen of the glands, while the basal membrane faces the exterior of the gland. A coronal view of the salivary glands revealed a clear F-actin cytoskeleton outlining each cell (Fig. 2c). However, we found that this cortical F-actin cytoskeleton was dismantled at the end of larval development. At −8 h PF, phalloidin staining showed tight bundles of cortical F-actin (Fig. 2d). Lifeact-Ruby, a fluorescently tagged peptide that binds actin^28, 29^, also showed that most of the actin within the cell at −8 h PF is present at the cortex (Fig. 2e). These phalloidin-stained F-actin bundles became less distinct at −4 and −1 h PF, and disappeared by PF (Fig. 2d). Consistently, Lifeact-Ruby imaging showed that this loss of cortical F-actin was associated with a gradual redistribution of actin from the cortex to the cytoplasm (Fig. 2e). This dissolution of F-actin appeared to start at −4 h PF, when the decrease in phalloidin staining was accompanied by increased Lifeact-Ruby signal at the cortex, as well as an increase in Lifeact-Ruby signal in the cytoplasm (*cf*. Lifeact-Ruby vs. phalloidin staining; Fig. 2d, e). These results reflect a rapid disassembly of cortical actin filaments into cytoplasmic monomers beginning at −4 h PF, at the peak of cortical caspase activation.

We next tested if cortical F-actin breakdown depended on the sublethal pulse of *rpr* and subsequent caspase activation at this stage. Indeed, loss of *rpr* function blocked dismantling of the F-actin cytoskeleton (Fig. 2f). Moreover, F-actin disassembly required *dronc* and *dcp-1* but not *drice* (Fig. 2f), mirroring the caspase cascade required for cortical staining of anti-cD1 during this stage (Fig. 1c). To further validate the role of caspases in F-actin disassembly, we tested the effect of inhibitors of caspase activation. Salivary gland-specific expression of *diap1*, the primary *Drosophila* IAP, disrupted F-actin breakdown, while expression of *p35*, the Baculovirus-derived effector caspase inhibitor, slightly inhibited this process (Fig. 2g). Importantly, salivary gland-specific RNAi knockdown of *rpr* or *dronc* strongly inhibited F-actin disassembly (Fig. 2g). These results indicate a cell-autonomous requirement for *rpr*-dependent antagonism of *diap1* and activation of *dronc* during F-actin breakdown in the larval salivary glands.

Given that the small pulse of *rpr* is dependent on ecdysone signaling, we also tested if ecdysone directly triggered the loss of cortical F-actin in salivary glands. We found that cell-autonomous disruption of ecdysone signaling through expression of the dominant negative *EcR* ^*F645A*^ in salivary glands blocked the loss of F-actin (Supplementary Fig. 3a). Conversely, addition of ecdysone to ex vivo cultures of salivary glands dissected at −8 h PF was sufficient to trigger F-actin breakdown, while addition of the translational inhibitor cycloheximide prevented ecdysone-triggered F-actin breakdown (Supplementary Fig. 3b), suggesting that translation of ecdysone-induced transcripts is critical for dismantling of cortical F-actin. Taken together, these results indicate that an ecdysone-induced sublethal pulse of *rpr* triggers caspase activation at the cortex, which, in turn, initiates dismantling of the cortical F-actin cytoskeleton.

### F-actin breakdown requires *dronc* localization and activity

**Fig. 3:**
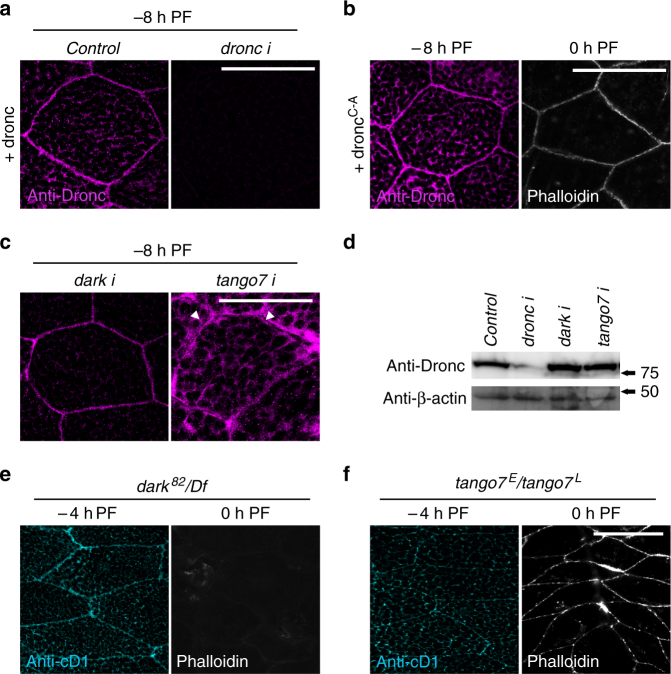
*Tango7* is required for cortical localization and activity of Dronc in living salivary glands. **a** Dronc localizes to the cortex of salivary glands at the end of larval development. Immunofluorescent staining using an antibody to detect Dronc protein (anti-Dronc, in *magenta*) in salivary glands overexpressing *dronc* under control of *Sgs3-GAL4* ( + *dronc*) shows that Dronc localizes to the cortex of salivary gland cells at −8 h PF. Co-expression of *dronc-RNAi* abolishes anti-Dronc staining. **b** F-actin dismantling requires Dronc catalytic activity. Anti-Dronc staining in salivary glands overexpressing a catalytically inactive Dronc ( + *dronc* ^*C-A*^, in *magenta*) under control of *Sgs3-GAL4* reveals that Dronc^C-A^ still localizes to the cortex at −8 h PF. However, phalloidin staining, in *white*, shows that F-actin does not break down in these glands at 0 h PF. **c** *tango7*, not *dark*, regulates cortical localization of Dronc. Anti-Dronc staining, in *magenta*, in salivary glands overexpressing *dronc* under control of *Sgs3-GAL4* shows that Dronc still localizes to the cortex upon co-expression of RNAi against *dark*. In contrast, *tango7* RNAi knockdown causes Dronc to diffuse away from the cortex toward the cytoplasm, with clear gaps lacking anti-Dronc staining visible at the cortex (*white arrowheads*). The cytoplasm at this stage is filled with secretory granules (*black* “spots”). **d** Dronc levels are not reduced in *tango7*-RNAi salivary glands. Western blot analysis of Dronc protein expression levels in *dronc-RNAi*, *dark-RNAi*, and *tango7-RNAi* salivary glands (under control of *Sgs3-GAL4*) shows that Dronc levels are dramatically reduced upon overexpression of *dronc-RNAi*, but are not decreased upon expression of *dark-RNAi* or *tango7-RNAi*. Salivary glands were dissected at −8 h PF; β-actin used as loading control. **e**, **f** *tango7*, not *dark*, is required for cortical caspase activation and dismantling of F-actin. **e** Cortical anti-cD1 staining, in *cyan*, is not disrupted in *dark* mutant salivary glands at −4 h PF. Phalloidin staining, in *white*, shows that F-actin breaks down normally in *dark* mutant salivary glands. **f** In contrast, cortical anti-cD1 staining is abolished at −4 h PF and F-actin fails to break down at 0 h PF in *tango7* mutant salivary glands. *Scale bars* represent 100 µm. Df, deficiency, PF, puparium formation

Our data thus far suggests that *rpr* activates caspases at the cortex, and it is this cortically restricted activity that breaks down the F-actin cytoskeleton. To understand the mechanisms that restrict caspase activity to the salivary gland cell cortex, we first examined the subcellular localization of Dronc protein. Staining with an anti-Dronc antibody showed that Dronc protein is enriched at the cortex at −8 and −4 h PF (Supplementary Fig. 4a). Accordingly, overexpression of full-length Dronc showed preferential targeting to the cortex at −8 and −4 h PF, but this cortical localization disappeared at PF (Fig. 3a; Supplementary Fig. 4b). Salivary gland-specific expression of *dronc-RNAi* abolished all Dronc staining at −8 h PF (Fig. 3a), confirming both the specificity of the antibody and efficiency of RNAi knockdown. Dronc protein localized to the cortex before induction of the *rpr* pulse that triggers cortical cleaved-Dcp-1 activation, suggesting that subcellular targeting of the initiator caspase may be responsible for restricting the *rpr*-initiated caspase cascade to the cell cortex. We next wanted to test the role of Dronc activity in F-actin remodeling, as caspases have previously been shown to regulate other non-apoptotic functions independently of their catalytic activity^30, 31^. Overexpression of Dronc accelerated the kinetics of F-actin breakdown in response to the small *rpr* pulse; in contrast, overexpression of a catalytically inactive *dronc* (*dronc* ^*C-A*^) prevented the breakdown of cortical F-actin (Fig. 3b). *dronc* ^*C-A*^ still localized appropriately (Fig. 3b), suggesting that it may inhibit F-actin breakdown by displacing endogenous Dronc from the cortex. Taken together, these results demonstrate that both cortical localization and activity of Dronc are essential for F-actin dismantling during salivary gland development.

### *tango7* not *dark* regulates *dronc* localization and activity

We next wanted to determine how Dronc is localized to the cell cortex. Given that initiator caspases require adaptor proteins for activation and function^3, 5, 32^, we tested if the Apaf-1 adaptor protein *dark*, which is the canonical regulator of *dronc* activity in *Drosophila* ^33, 34^, was required for localization and/or function of *dronc* at the cortex. To our surprise, knockdown of *dark* had no effect on the cortical localization of Dronc protein (Fig. 3c). Moreover, *dark* null mutant glands still showed cortical anti-cD1 staining, and dismantling of cortical F-actin occurred normally (Fig. 3e). These results demonstrate that *dark* is not required for the localization or function of *dronc* at the salivary gland cell cortex. To identify other potential adaptor proteins for *dronc* in salivary glands, we conducted an RNAi-based screen among candidate proteins known to physically interact with *dronc* ^35–38^. The strongest suppressor of F-actin breakdown in our screen was *tango7* (Supplementary Fig. 5a). Salivary gland-specific knockdown of t*ango7* also disrupted cortical Dcp-1 activation (Supplementary Fig. 5b, c), indicating that *tango7* plays a cell-autonomous role in caspase-dependent F-actin dismantling. *tango7* mutant glands also disrupted both the cortical activation of Dcp-1 and the dismantling of cortical F-actin (Fig. 3f). Consistent with a function in F-actin breakdown, we found that Tango7 protein localized to the salivary gland cell cortex. Analysis of Tango7 localization using recently generated endogenous superfolder GFP (sGFP)-tagged *tango7* ^39^ showed that Tango7 protein displayed subcellular dynamics similar to Dronc, with robust cortical localization at −8 h PF that was lost at 0 h PF (Supplementary Fig. 5d, e). This places Tango7 protein at the right time and place to function with Dronc in F-actin dismantling. We also investigated the effect of *tango7* knockdown on Dronc protein stability, as previous studies have shown that loss of *tango7* reduces Dronc protein levels^37^. However, western blot analysis of Dronc protein levels showed that knockdown of *tango7* did not reduce Dronc expression in salivary glands (Fig. 3d). Importantly, *tango7* appeared to be critical for proper cortical localization of Dronc. In *tango7* knockdown glands, Dronc protein no longer displayed tight cortical localization and appeared instead to diffuse towards the cytoplasm, with conspicuous gaps lacking Dronc protein at the cortex (Fig. 3c). Taken together, these results demonstrate that *tango7* is required for localization, activation, and function of *dronc* in F-actin dismantling at the salivary gland cell cortex.

### Caspase activation in mutually exclusive subcellular domains

**Fig. 4:**
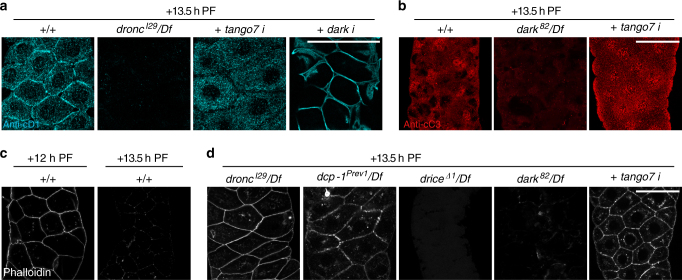
Subcellular domains of caspase activation are independently regulated in dying salivary glands. **a** *tango7* and *dark* regulate mutually exclusive subcellular domains of caspase activation in dying glands. Anti-cD1 staining, in *cyan*, is visible at both the cortex and in the cytoplasm of control salivary glands at + 13.5 h PF; both cortical and cytoplasmic staining is lost in *dronc* mutant salivary glands. Salivary gland-specific knockdown of *tango7* (with *Sgs3-GAL4*) does not affect cytoplasmic anti-cD1, but disrupts cortical anti-cD1. In contrast, salivary gland-specific knockdown of *dark* (with *fkh-GAL4*) disrupts cytoplasmic anti-cD1 but not cortical anti-cD1. **b** Cleaved caspase-3 (anti-cC3, in *red*), a diagnostic marker of caspase activation and apoptosis, does not require *tango7*. Anti-cC3 staining is visible only in the cytoplasm of control glands at + 13.5 h PF; this staining is lost in *dark* mutant animals. However, anti-cC3 staining is unaffected upon salivary gland-specific RNAi knockdown of *tango7* (using *Sgs3-GAL4*). **c** Cortical F-actin is dismantled in dying salivary glands. The cortical F-actin cytoskeleton is intact in control salivary glands at + 12 h PF, just prior to the death response (phalloidin, in *white*). However, phalloidin staining shows that F-actin is dismantled at + 13.5 h PF. **d** F-actin dismantling during the death response requires *dronc*, *dcp-1*, and *tango7*. Phalloidin staining, in *white*, indicates that F-actin breakdown is disrupted in *dronc* and *dcp-1* mutant and *tango7-RNAi* (*Sgs3-GAL4*) salivary glands at + 13.5 h PF, but F-actin breakdown is unaffected in *drice* and *dark* mutant salivary glands. *Scale bars* represent 100 µm. Df, deficiency, PF, puparium formation

Both *dronc* and *dark* play a critical role in caspase activation during the death response in salivary glands^40–42^, about half a day after the events discussed so far. To further examine the roles of *tango7* and *dark* in the activation of *dronc*, we tested the role of these adaptors during the death response in salivary glands. At this later stage, anti-cD1 staining still showed robust signal at the cortex, but now also showed significant signal in the cytoplasm (Fig. 4a). Analysis of a timecourse of anti-cD1 staining showed that cortical activation is short-lived at this stage, while the cytoplasmic activation persisted longer (Supplementary Fig. 6), suggesting that cortical activation may be a transient event in response to the high-magnitude induction of *rpr*. Loss of *dronc* disrupted all anti-cD1 staining (Fig. 4a), indicating that Dcp-1 activation in both the cortical and cytoplasmic subcellular domains requires *dronc* activity. Strikingly, salivary gland-specific knockdown of *tango7* disrupted anti-cD1 staining at the cortex without affecting anti-cD1 staining in the cytoplasm (Fig. 4a). Conversely, RNAi knockdown of *dark* disrupted anti-cD1 staining in the cytoplasm but not at the cortex (Fig. 4a). Consistent with previously published results, *dark* was required for anti-cC3 staining at this stage, which appeared to be only cytoplasmic (Fig. 4b). In contrast, knockdown of *tango7* did not appear to affect cytoplasmic anti-cC3 staining (Fig. 4b), although *tango7* knockdown appeared to disturb normal morphological breakdown during the death response. Anti-cC3 staining has previously been reported to detect *dronc* activity^43^, but our results suggest that this antibody specifically detects *dark*-dependent *dronc* activity in *Drosophila* tissues. Taken together, these results demonstrate that *tango7* is only required for caspase activation at the cortex, while *dark* is required for caspase activation in the cytoplasm. Importantly, activation of caspases in one subcellular domain does not appear to affect activation of caspases in the other domain, suggesting that caspase activation within these subcellular domains is restricted and compartmentalized.

We next tested if subcellular domain-specific activation of caspases resulted in compartment-specific functions. Cortical F-actin, which is transiently dismantled after the small pulse of *rpr* at PF, was dismantled once again during the death response (Fig. 4c). Similar to F-actin dismantling at PF, this second dismantling of cortical F-actin in dying glands also required *dronc* and *dcp-1*, but not *drice* (Fig. 4d), demonstrating that a similar caspase cascade is responsible for the cortical function of caspases in both living and dying cells. Importantly, knockdown of *tango7* blocked breakdown of cortical F-actin in dying glands, despite the robust activation of caspases in the cytoplasm (Fig. 4a, b, d). Conversely, although loss of *dark* blocked activation of caspases in the cytoplasm (Fig. 4b), absence of *dark* did not disrupt *dronc*-dependent dismantling of cortical F-actin (Fig. 4d). Thus, contrary to commonly held assumptions, cytoplasmic activation of caspases does not result in widespread cellular caspase activity; instead, *tango7* and *dark* appear to define mutually exclusive subcellular domains of *dronc* activation and function in dying salivary glands.

### *tango7* is required for *dronc*-dependent dendrite pruning

**Fig. 5:**
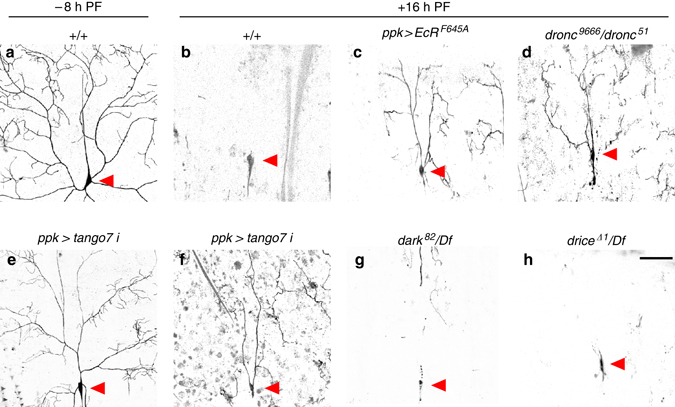
Caspase-dependent pruning of class IV ddaC dendrites during metamorphosis requires *tango7* but not *dark*. **a**, **b** Dendritic arborizations, visualized by *ppk-GAL4*-driven expression of GFP, are present at −8 h PF **a**, but are completely pruned by + 16 h PF **b**. **c**, **d** Expression of a dominant-negative ecdysone receptor (*EcR* ^*F645A*^) using *ppk-GAL4* (**c**) or mutation of *dronc* (**d**) disrupts pruning at + 16 h PF. **e**, **f** Fewer dendritic arborizations form at −8 h PF upon RNAi knockdown of *tango7* (using *ppk-GAL4*) **e**; however, these branches are not properly pruned at + 16 h PF **f**. **g**, **h** Dendritic pruning occurs normally in *dark* (**g**) or *drice* (**h**) mutant animals. *Red arrowheads* indicate the soma of ddaC neurons. *Scale bars* represent 100 µm. Df, deficiency, PF, puparium formation

To determine the extent to which *tango7*-dependent control of *dronc* activity is used in other physiological contexts, we examined the role of *tango7* in *dronc*-dependent pruning of dendritic arborizations during metamorphosis. Dendrites of the class IV ddaC sensory neurons are pruned in an ecdysone-dependent manner, and this pruning involves remodeling of the cortical F-actin cytoskeleton^16, 44–46^. We found, consistent with earlier reports^16, 17, 44, 45^, that blocking ecdysone signaling or loss of *dronc* disrupted dendritic pruning (Fig. 5a–d); however, in contrast to a previous report^16^, we found that pruning occurred normally in *dark* mutant animals (Fig. 5g). Our result is consistent with axonal pruning in mammals, which requires caspase-9 activity, but not Apaf-1^14^. We also found that loss of *drice* did not block dendrite pruning (Fig. 5h), while knockdown of *tango7* did (Fig. 5e, f). Although ddaC sensory neurons formed fewer dendritic branches upon knockdown of *tango7*, these branches were not properly pruned. These results demonstrate that the caspase cascade and regulatory mechanisms we defined in larval salivary glands also function during dendrite pruning, suggesting that the *tango7*-dependent pathway outlined here may represent a conserved and widely utilized mechanism for regulating cortical functions of caspases.

### Caspase-dependent control of elasticity and timely secretion

**Fig. 6:**
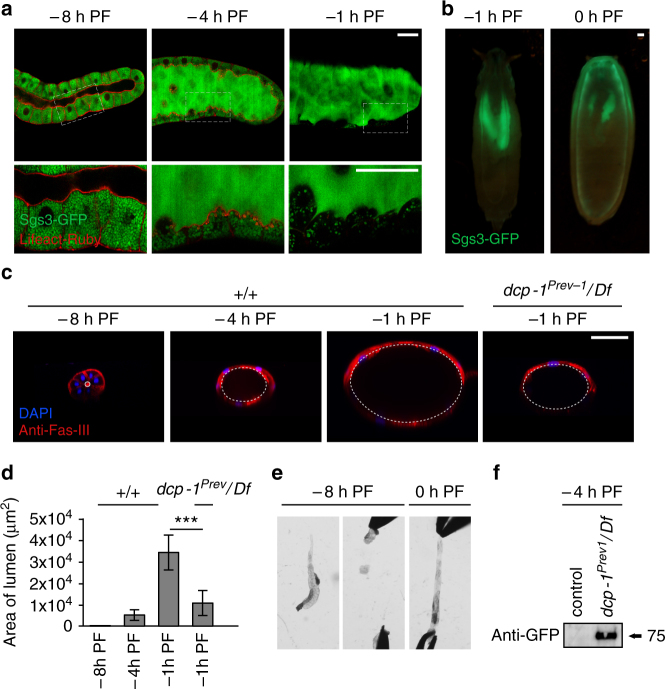
Cortical caspase activation in living salivary glands controls tissue elasticity during expulsion of mucin-like glue proteins. **a** Dismantling of cortical F-actin in acinar cells coincides with secretion of glue proteins; *boxed areas* imaged at higher magnification below. At −8 h PF, glue proteins (Sgs3-GFP, in *green*) are present only in the cells of the salivary gland. At −4 h PF, exocytosis of glue proteins from cells into the lumen begins; by −1 h PF, all glue is present in the lumen. Lifeact-Ruby, in *red*, shows that F-actin begins to break down at −4 h PF, and dismantling is nearly complete by −1 h PF. Salivary glands were imaged live/unfixed. **b** Glue proteins present in the lumen at −1 h PF are expelled onto the surface of the animal at 0 h PF. **c**, **d** The lumen increases dramatically in size during glue exocytosis, and luminal expansion requires caspase activity. **c** Transverse view of salivary glands shows luminal expansion during glue secretion. Fasciclin-3 staining (anti-Fas-III, in *red*) marks septate junctions, nuclei stained with DAPI in *blue*. At −8 h PF, salivary glands have a narrow lumen; however, as glue exocytosis progresses, the lumen expands dramatically until reaching maximal size at −1 h PF. The lumen of *dcp-1* mutant salivary glands does not expand normally. **d** Quantification of luminal area for stages and genotypes shown in **c** (*n* = 10 per timepoint, *error bars* indicate s.d., asterisks indicate *p* < 0.01 determined by one-tailed *t*-test). **e** Salivary gland elasticity is developmentally controlled. At −8 h PF, salivary glands are rigid and tear at the slightest pull. In contrast, 0 h PF salivary glands can be stretched beyond their normal length. Equal force was applied to each stage; *n* = 20 tested per stage. **f** Luminal expansion is critical for timely expulsion of glue proteins. Glue expulsion normally occurs after larvae become stationary. Control wandering larvae at −4 h PF do not expel glue, while *dcp-1* mutant larvae precociously expel glue at this stage. Expelled glue proteins (in Sgs3-GFP animals) were collected and measured by western blot with anti-GFP antibodies. *Scale bars* represent 100 µm. Df, deficiency, PF, puparium formation

In dying salivary glands, cortical F-actin is likely dismantled as a step toward cellular demolition; however, the function of F-actin breakdown in glands at the end of larval development was unclear. To understand why the cortical F-actin cytoskeleton is dismantled at the end of larval development, we first examined the most conspicuous function of glands at this stage: the secretion of mucin-like “glue” proteins. Glue secretion occurs in two discrete steps: first, exocytosis of glue-containing vesicles from acinar cells to the lumen (Fig. 6a); and second, expulsion of glue proteins from the lumen to the exterior, allowing the soon-to-be stationary pupa to adhere to a solid surface (Fig. 6b). Exocytosis of glue-containing vesicles has recently been shown to require the actin cytoskeleton^47, 48^. However, imaging and western blot analysis showed that glue exocytosis occurred normally in *dronc* mutant glands (Supplementary Fig. 7), indicating that *dronc*-dependent breakdown of cortical F-actin is not required for this step of glue secretion.

Exocytosis of glue proteins results in a dramatic morphological change in the salivary glands. By −1 h PF, glue exocytosis is nearly complete, but expulsion from the lumen onto the animal surface has not yet occurred. As a result, all of the secretory content is held within the lumen of the glands (Fig. 6a). Glue proteins are hygroscopic mucins which increase considerably in volume upon contact with water; therefore, the exocytosis and accumulation of secretory content resulted in a dramatic expansion of the lumen of the salivary glands (Fig. 6c, d). Luminal expansion began at −4 h PF: the same time as F-actin breakdown, raising the question of whether there is a functional relationship between these two processes. We found that mutants of the ecdysone primary response gene *BR-C* (*rbp* ^5^) produce very few glue-containing vesicles; however, the vesicles that are produced are secreted normally. A reduced load of secretory content results in only slight luminal expansion in these glands, enabling a more detailed analysis of F-actin breakdown. Importantly, we found that F-actin is still dismantled in *rbp* ^5^ mutant salivary glands (Supplementary Fig. 8a), indicating that F-actin does not passively break as a result of luminal expansion. Additionally, high magnification analysis reveals clear “fraying” of the F-actin structure at −1 h PF, causing F-actin to drop away from the cortex; this fraying is accompanied by complete dissolution of the F-actin meshwork (Supplementary Fig. 8b). Importantly, the fraying of F-actin is blocked in *rbp* ^5^ mutant salivary glands upon overexpression of *diap1* or RNAi knockdown of *rpr* or *tango7*, further confirming that this process is caspase-dependent (Supplementary Fig. 8c), and suggesting that caspases may sever F-actin branchpoints during the breakdown process. Taken together, these results demonstrate that F-actin dismantling is an independently regulated process during salivary gland development.

We next tested if F-actin played a role in the second step of glue secretion: expulsion from the lumen onto the surface of the animal. Interestingly, the lumen of *dcp-1* mutant or *tango7* knockdown glands, which both failed to dismantle cortical F-actin, did not expand upon glue exocytosis (Fig. 5c, d; Supplementary Fig. 9), raising the possibility that F-actin dismantling facilitates “stretching” of the glands to accommodate increasing amounts of secretory content in the lumen. Consistently, at PF, when all cortical F-actin is gone, wild type salivary glands were extremely “elastic” and could easily be stretched well beyond their normal length (Fig. 5e). In contrast, glands at −8 h PF, with an intact cortical F-actin cytoskeleton, tore when pulled (Fig. 5e). Finally, *dcp-1* mutant salivary glands precociously expelled their secretory content prior to the onset of metamorphosis (Fig. 5f), indicating that the retained rigidity of the F-actin cytoskeleton in these animals did not permit luminal expansion to accommodate the large volume of secreted glue proteins. These results highlight a novel aspect of exocrine biology, in which caspase-dependent remodeling of cortical F-actin in acinar cells regulates tissue elasticity and facilitates storage of luminal content prior to its timely expulsion.

## Discussion

Principles that govern the activation and function of caspases have fallen short in providing an understanding for how these enzymes can be activated to perform both delicate intracellular remodeling in living cells and total destruction in dying cells. In this paper, we provide new insights into the mechanisms that regulate caspase activation by comparing two completely different biological outcomes in the same tissue that both require caspase function. We show that the *Drosophila* homolog of caspase-9, *dronc*, is required for dismantling of the cortical F-actin cytoskeleton during salivary gland development—a role that is distinct from its known function in the salivary gland death response during metamorphosis. By systematically dissecting the regulation of *dronc* function at the cortex, we show that cortical functions of *dronc* are regulated independently from its cytoplasmic functions. The cytoplasmic functions of activated *dronc* require the canonical adaptor protein *dark*, while the cortical roles of *dronc* require *tango7*. In this manner, *tango7* and *dark* restrict the function of *dronc* to distinct subcellular domains. Moreover, we also show that these two functions can be initiated independently through differential amplification of IAP antagonist expression, providing a model for how lethal and vital roles of caspases can be differentially activated in the same cell. Finally, we identify a new non-apoptotic function for caspases in the control of tissue elasticity to accommodate buildup of secreted products in the lumen of secretory tissues, facilitating their timely release.

Our results demonstrate that caspases can be activated in distinct, mutually exclusive subcellular domains within a single cell, and that these subcellular domains are generated by use of unique caspase adaptor proteins. Local activation of caspases, as detected by staining with antibodies to activated caspases, has been reported before;^16–18, 20, 49^ however, here we demonstrate that local activation is achieved by targeting caspases to subcellular domains, and this targeting is necessary for subcellular functions of these caspases. Importantly, we show that caspases can be activated specifically in one domain without being activated in another, providing a mechanism that allows control of caspase activity with a previously unknown level of subcellular precision. However, the mechanisms that restrict caspase cascades to distinct subcellular compartments remain unclear. It is possible that caspase expression levels are intentionally kept low during non-lethal responses, and localized enrichment mediates subcellular domain-specific activation. For example, if most of the Dronc protein present in the cell localizes to the cortex, then this specific localization may restrict caspase functions to the cortical compartment. This model fits with our results at the end of larval development; however, in dying glands, caspases are independently activated in cortical and cytoplasmic compartments, suggesting that additional mechanisms are in play to restrict caspase activity to the appropriate subcellular compartment. For example, it is possible that caspase cascades occur within a physical complex consisting of initiator caspases, their adaptor proteins, effector caspases, and their substrates. In this model, only one of these proteins, likely the initiator caspase, would need to be subcellularly localized in order to generate a compartment-specific caspase cascade. However, resolution of this possible mechanism will require further studies. This subcellular domain-specific model for caspase activation contrasts with the commonly held belief that activated caspase cascades passively perpetuate themselves and spread throughout the cell, and also opens the possibility that caspases, through specific subcellular localization mediated by adaptor proteins, may play a role in many yet-to-be-identified biological processes.

We demonstrate that differential amplification of IAP antagonists at specific developmental stages determines lethal vs. non-lethal outcomes of caspase activation. In our system, differential amplification is accomplished through the use of transcription factors that function downstream of a steroid hormone signal. However, caspases must have an ability to “sense” the magnitude of the IAP antagonist pulse, ensuring that they initiate the appropriate lethal or non-lethal responses. One possible “sensing” mechanism may involve the aforementioned selectivity of initiator caspase adaptor proteins, like we observed with *tango7* and *dark*. In this model, some adaptor protein complexes would require a lower IAP antagonist threshold for initiator caspase activation than others. However, elucidation of the detailed molecular mechanisms mediating “sensing” of IAP antagonist expression levels will require further study. Finally, our results indicate that small pulses of IAP antagonist expression are tissue specific, raising the possibility that many more of these pulses are generated in other tissues and developmental stages that have not yet been detected or characterized. Our data suggests that non-lethal, physiological functions of caspases may be more widespread than previously thought.

Our results show that caspases play a novel role during the secretion of glue proteins. Glue proteins are essential to allow a newly formed prepupa to adhere to a solid surface; however, when cortical F-actin dismantling fails, glue precociously “leaks” onto the surface of the animal. Although precocious expulsion of glue does not appear to have a deleterious effect in the lab, in the wild, it may adversely affect fitness by inhibiting larval movement or reducing the ability of the animal to stick securely to a surface during metamorphosis. Additionally, our results raise the question of whether other exocrine tissues in different species, such as the mammary gland, may utilize caspases in a similar manner to accommodate large amounts of secreted luminal products prior to their release.

In conclusion, systematic analysis of vital and lethal responses to caspase activation in the same cells has revealed mechanisms that allow caspases to be activated without killing the cell. Our results demonstrate that caspases can be activated in mutually exclusive subcellular domains, where activation of caspases in one domain does not trigger activation of caspases in another domain. We show that these subcellular domains are generated by different caspase adaptor proteins. It is likely that yet-to-be-identified adaptor proteins define other subcellular domains and, in so doing, help regulate the many physiological functions of caspases. Moreover, our results demonstrate that some of these subcellular domains have lower thresholds for activation of caspases, thereby allowing sublethal pulses of IAP antagonists to selectively initiate physiological functions of caspases. Together, these results outline a simple conceptual framework for controlling caspase activation during normal development and physiology.

## Methods

### Fly strains and genetics

The following strains were obtained from the Bloomington Drosophila Stock Center: Sgs3-GFP, Dronc^51^, UAS-Lifeact-Ruby, Sgs3-Gal4, UAS-tango7-RNAi, E74A^neo24^, br^rpb5^, UAS-EcR^F645A^, UAS-CD8-GFP, fkh-Gal4, ppk-Gal4, Df(3L)Exel6112 (for med24), Df(3L)81k19 (for E74A), Df(3R)BSC547 (for drice), Df(2R)BSC359 (for dark), Df(2R)BSC785 (for dcp-1), Df(3L)BSC282 (for dronc) and Df(3L)H99 (for rpr). The Vienna Drosophila RNAi Center provided Tango7-sGFP and the following RNAi lines: UAS-dronc-RNAi, UAS-dark-RNAi, UAS-rpr-RNAi. Colleagues in the fly community provided the following stocks: dronc^I24^ and dronc^I29^ ^50^, drice^∆1^ ^51^, dark^82^ ^42^, dcp-1^Prev1^ ^52^, reaper^87^ ^53^, UAS-dronc^C-A^ ^54^, tango7^E^ and tango7^L^ ^38^, med24^psg5^ ^26^, UAS-dronc-GFP^34^. All crosses were performed at 25 °C in temperature-controlled incubators. Salivary gland-specific overexpression or RNAi knockdown was performed with the Sgs3-Gal4 driver unless otherwise noted. Wherever appropriate, control genotypes included an additional UAS transgene (UAS-GFP) to facilitate comparisons with experimental genotypes.

### Developmental staging

All animals were raised at 25 °C on cornmeal molasses media with granulated yeast. For staging third instar larvae, we combined the standard “blue food” technique^55^ with use of the mid-third instar transition-specific reporter, Sgs3-GFP^56^. For −8 h PF larvae, animals with “light blue” guts without glue secretion were collected. For −4 h PF, animals with “clear blue” guts and glue in the lumen were selected. Finally, for −1 h PF, we collected “clear” gut animals that were stationary with everted spiracles and soft cuticles. These staging methods have been independently tested for timing to PF. For animals after PF, animals were collected at PF and aged for the appropriate time at 25 °C on a dampened filter paper plate. For stages at 12 h PF or later, we collected animals that were synchronized at head eversion (equivalent to 12 h PF) and aged for the appropriate length of time thereafter.

### Quantitative RT-PCR

mRNA expression levels of target genes were measured using quantitative real time PCR (qPCR). RNA was isolated from tissues dissected from appropriately staged animals using the RNeasy Plus Mini Kit (Qiagen). cDNA was synthesized from 400 ng of total RNA using the SuperScript III First-Strand Synthesis System (Invitrogen). qPCR was performed on a Roche 480 LightCycler using LightCycler 480 SYBR Green I Master Mix (Roche). The target gene primer sequences used in this study were previously validated: *rpr* ^57^, *hid* ^57^, *E74A* ^58^, and *rp49* ^59^. In all cases, samples were run simultaneously with three independent biological replicates for each target gene. *rp49* was used as the reference gene. To calculate changes in relative expression and error bars, we used the Relative Expression Software Tool (REST)^60^. REST calculates confidence intervals and *p*-values for relative expression values using integrated bootstrapping and randomization methods. Standard error is calculated based on a confidence interval centered on the median; therefore error bars calculated by REST reflect asymmetrical tendencies in the data.

### Immunofluorescent staining and image acquisition

Salivary glands dissected from appropriately staged animals were fixed for 30 min in PBS with 0.1% Triton X-100 (PBST) and 4% formaldehyde, blocked overnight with PBST/4% BSA, and stained with the appropriate primary and secondary antibodies diluted in PBST/4% BSA. All tissues were fixed and stained using identical conditions, and a wild type control was always done as a base line to detect any antibody signal loss. Identical microscope acquisition settings and image processing parameters were used for each experiment/set of paired samples. All primary antibodies were tested for consistency to reduce variability between lots, and the same lot was used for all experiments whenever possible. Primary antibodies used were: rabbit α-Cleaved Dcp-1 (1:200; Cell Signaling #9578, Lot 1), rabbit α-Dronc (1:200; gift from P. Friesen, University of Wisconsin-Madison), rabbit α-Cleaved Caspase-3 (1:200; Cell Signaling #9661), and mouse anti-Fasciclin-3 (1:50; Developmental Studies Hybridoma Bank 7G10). Secondary antibodies used were AlexaFluor 488 anti-mouse and anti-rabbit (1:200; Invitrogen A11029 and A11034) and anti-rabbit Cy3 (1:200; Jackson Immuno-Research Labs 715-165-150). For DNA staining, DAPI was used (1:1000; Invitrogen). For phalloidin staining, salivary glands were dissected in PBS, fixed with 4% formaldehyde in PBS for 30 min and stained in the dark with a 100 nM working solution of 488-conjugated Phalloidin (Life Technologies) for 30 min. Stained tissues were mounted in Vectashield (Vector Laboratories). All images were captured on an Olympus FluoView FV1000 confocal microscope and optimized with FV10-ASW software. Images shown in figures are representative of at least three independent experiments with *n* ≥ 10 tissues analyzed per experiment. Whenever possible, results were confirmed by an independent lab member who was blinded to the genotype/condition being analyzed. For 3D volume views, stained salivary glands were mounted in a 35 mm glass-bottom dish (MatTek Corporation) in 50 °C 0.8% agarose and imaged immediately using a *Z-*stack sequence at 3 µm intervals on a Nikon A1R confocal microscope. Volume views were optimized using the Nikon NIS Element software, and lumen size quantification was done using ImageJ. Light microscope images of whole salivary glands were taken on an Olympus SZX16 stereomicroscope using an Olympus DP72 digital camera with DP2-BSW software.

### Ex vivo salivary gland cultures

Ex vivo cultures were performed using standard methods^61^. Salivary glands were dissected in oxygenated Schneider’s insect media (Sigma) and pre-incubated for 30 min in a “lung” with flowing oxygen. Fresh oxygenated media with 5 µM of 20-hydroxyecdysone (Sigma) and/or 85 µM cycloheximide (Sigma) was added to the appropriate samples. Salivary glands were stained and imaged as described above.

### Glue secretion assays

For glue protein imaging, salivary glands from strains carrying the Sgs3-GFP fusion protein were dissected in PBS and immediately imaged live on an Olympus FluoView FV1000 confocal microscope. For glue secretion western blot assays, three appropriately staged animals carrying Sgs3-GFP were collected, washed with water, and the glue content that had not been expelled was measured. The animals were homogenized in 50 µl of hi-salt lysis buffer (25 mM HEPES pH 7.4, 300 mM NaCl, 1.5 mM MgCl_2_, 1 mM EDTA, 0.5% Triton X-100, 50 mM β-glycero-phosphate, 50 mM NaF, 1 mM Na_3_V0_4_, 5 mg/ml Pepstatin A, 1 mM DTT, 5 mg/ml Aprotinin, 200 mM PMSF, 10 mg/ml Leupeptin, 4 nM Microsystin). In total, 10 µl of each sample was then subjected to a western blot analysis. To monitor premature expulsion, five wandering larvae (at −8 h PF) of the appropriate genotype were placed in small PCR tubes, allowed to wander for 2 h, and then removed. Only samples lacking animals that had started PF were used; 20 µl 2× sample buffer (1.0 M Tris-HCl pH 6.8, 8% SDS, 40% Glycerol, 20% 2-Mercaptoethanol) was added to the small PCR tubes and vortexed vigorously to collect expelled proteins. The samples were heated at 95 °C for 5 min and were subjected to a western blot analysis.

### Western blot analysis

Protein expression levels were measured by western blot analysis. Primary antibodies used were rabbit anti-GFP (1:2000; Torrey Pines TP401), rabbit anti-Dronc (1:1000; gift from P. Friesen, University of Wisconsin-Madison), mouse anti-β-tubulin (1:1000; Millipore #05-661), and rabbit anti-β-actin (1:1000; Cell Signaling #4967). Secondary antibodies used were alkaline phosphatase-conjugated goat anti-rabbit and anti-mouse IgG (1:30,000; Sigma A3687 and A3438). Membranes were developed for imaging with ECF substrate (GE Healthcare) and were imaged using a Storm 840 Scanner (Amersham Bioscience) and processed with ImageQuant TL software version 7.0 (GE Healthcare). Full-length, uncropped blots are included in Supplementary Fig. 10.

### Dendrite pruning

Dendritic arborizations of the ddaC neurons on the larval abdomen were imaged by expression of *CD8-GFP* driven by *ppk-Gal4* ^46^. For −8 h PF, the larvae were placed in a drop of PBS and pushed between a coverslip and microscope slide until the animals were immobile but still alive. For 16 h PF, the cuticle of appropriately staged pupae was carefully removed and the animals were mounted in PBS. All mounted samples were imaged immediately on an Olympus FluoView FV1000 confocal microscope with 10−25 optical sections at 1.5 µm intervals. Images were optimized using the FV10-ASW software and inverted using Photoshop CC 2015.

### Data availability

All relevant data is provided in the paper and supplemental supporting information.

## Electronic supplementary material

Supplementary Information
