# Supplementary material for: Tango7 regulates cortical activity of caspases during reaper-triggered changes in tissue elasticity
Source: Nat Commun. 2017 Sep 19;8:603. doi: 10.1038/s41467-017-00693-3 (PMC5605750; doi:10.1038/s41467-017-00693-3)
Supplement: Supplementary file 1 — Supplementary Information [file 41467_2017_693_MOESM1_ESM.pdf]

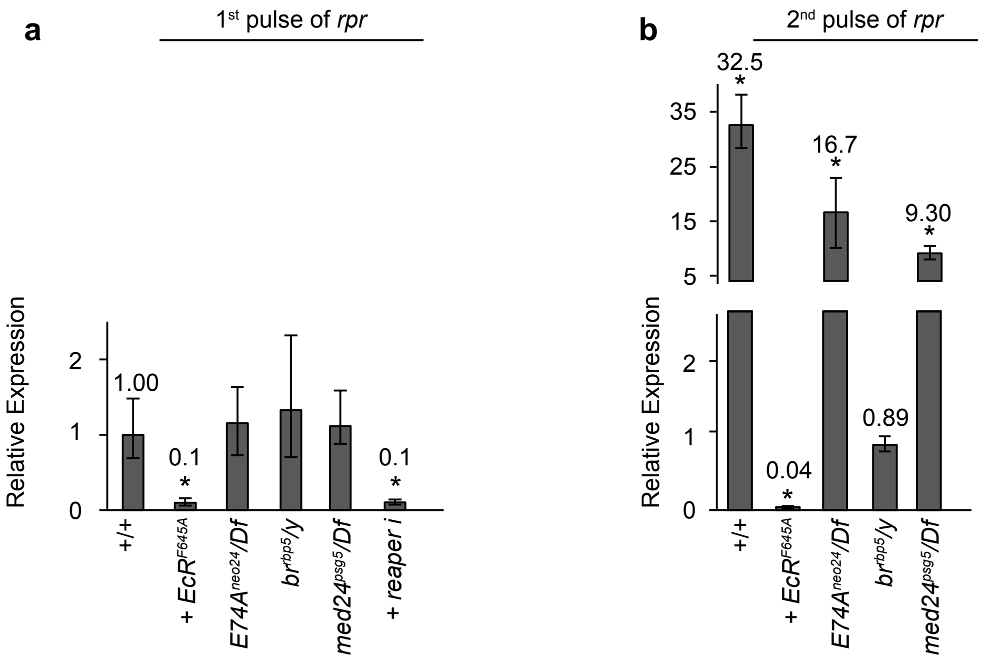

Supplementary Figure 1. Differential amplification of *reaper* (*rpr*) pulses in salivary glands. Genetic control of the two ecdysone-triggered pulses of *rpr* in salivary glands at the onset of metamorphosis. **(a)** The small pulse of *rpr* at the end of larval development depends on ecdysone but is independent of the canonical regulators of ecdysone-triggered transcription. qPCR analysis of *rpr* mRNA levels in salivary glands dissected at 0 h PF and normalized to *rpr* levels in controls. We chose to synchronize the animals at 0 h PF to avoid potential complications with developmental timing in different mutant backgrounds. Expression of either the dominant negative ecdysone receptor (*EcR*^{F645A}) or *rpr-RNAi* (using the salivary gland-specific *Sgs3-GAL4* driver) effectively disrupts the pulse of *rpr*. However, glands dissected from *E74A*, *BR-C*, or *Med24* mutant animals still induce *rpr* at levels comparable to control glands. **(b)** The larger pulse of *rpr* during programmed cell death, at +13.5 h PF, requires the canonical regulators of ecdysone signaling to amplify the transcriptional response. qPCR analysis of *rpr* mRNA expression from salivary glands dissected at +13.5 h PF. Data was normalized to the small pulse of *rpr* at -4 h PF in wild type glands (*i.e.* the second pulse is 32.5-fold larger than the first one). As with the first pulse, expression of *EcR*^{F645A} effectively disrupts the second pulse of *rpr*. Glands dissected from *E74A*, *BR-C*, and *Med24* mutant animals significantly reduce the induction of *rpr*; however, *rpr* is still induced at, or higher than, the levels of the first pulse (*y*-axis is split to facilitate comparison with the first pulse). *y*-axis shows relative expression; *x*-axis shows genotypes being analyzed. Expression for panel a shown relative to control at 0 h PF; panel b shown relative to -4 h PF; all samples normalized to the reference gene *rp49*. Three biological samples analyzed for each stage; error bars represent standard error determined by REST analysis (see Methods); asterisks indicate p-value <0.05 calculated by REST analysis. Df: Deficiency.

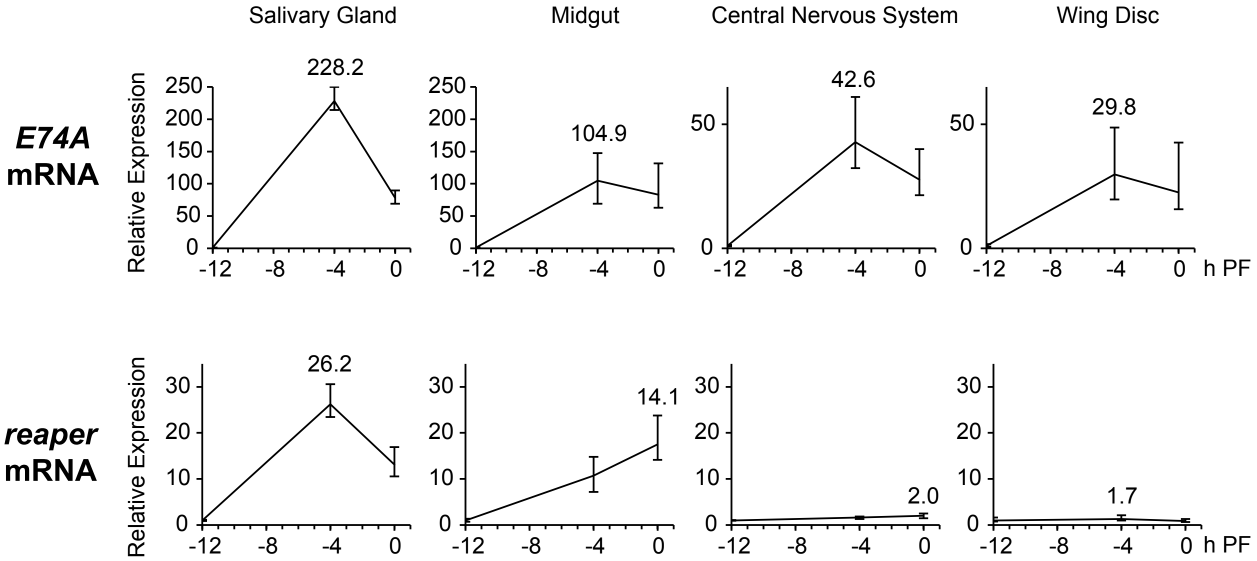

Supplementary Figure 2. Tissue-specific induction of *rpr* at the end of larval development. qPCR analysis of paired tissues (dissected from the same animals) at -12, -4, and 0 h PF. All examined tissues (salivary gland, midgut, central nervous system, wing disc) induce the ecdysone primary response gene *E74A*, indicating a robust ecdysone response. However, only the salivary glands and midgut induce *rpr* expression; no significant change in *rpr* levels is observed in the central nervous system or wing discs. The *y*-axis shows relative expression; the *x*-axis shows developmental stage in hours relative to puparium formation (PF). All samples were normalized to the reference gene *rp49*. Expression shown relative to levels at -12 h PF. Three biological samples analyzed for each stage; error bars represent standard error determined by REST analysis (see Methods). PF: Puparium Formation.

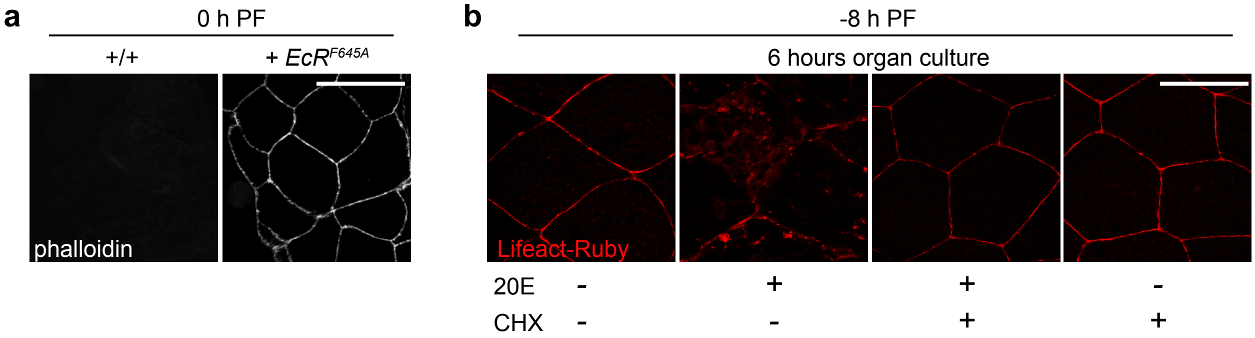

Supplementary Figure 3. Ecdysone is necessary and sufficient for dismantling of cortical F-actin in salivary glands at the end of larval development. **(a)** Ecdysone signaling is necessary for cortical F-actin breakdown. Phalloidin staining, in white, shows that F-actin is dismantled in control glands at puparium formation (0 h PF), but when ecdysone signaling is blocked by salivary gland-specific (*Sgs3-GAL4*) expression of a dominant negative ecdysone receptor (*EcR*^{F645A}), F-actin does not break down. **(b)** The steroid hormone ecdysone is sufficient to trigger cortical F-actin breakdown in *ex vivo* cultures. Salivary glands dissected at -8 h PF do not dismantle F-actin, shown by the actin marker Lifeact-Ruby in red, after 6 hours in *ex vivo* culture. Under the same conditions, addition of 20-hydroxyecdysone (20E, *a.k.a.* ecdysone) triggers F-actin dismantling. Co-culture of 20E with the translational inhibitor cycloheximide (CHX) prevents ecdysone-triggered dismantling of cortical F-actin, indicating that translation of ecdysone-induced transcripts regulates F-actin breakdown. Finally, cycloheximide alone does not dismantle the cortical F-actin cytoskeleton. All samples were cultured simultaneously and repeated in n>20; glands were fixed for imaging. Scale bars represent 100μm. PF: Puparium Formation; 20E: 20-hydroxyecdysone/ecdysone; CHX: cycloheximide.

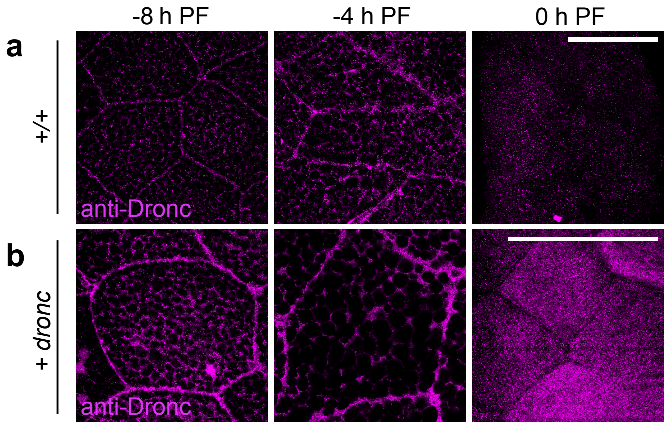

Supplementary Figure 4. Subcellular localization of Dronc in salivary gland cells. (a) Immunofluorescent staining for Dronc protein (anti-Dronc, in magenta) in wild type salivary glands shows that endogenous Dronc localizes to the cell cortex at -8 and -4 h PF; however, this cortical localization is lost at 0 h PF. **(b)** Immunofluorescent staining for Dronc protein (anti-Dronc, in magenta) in salivary glands overexpressing Dronc (+*dronc*; with *Sgs3-GAL4* driver) shows similar cortical localization of Dronc protein at -8 and -4 h PF; anti-Dronc staining is lost from the cortex and becomes cytoplasmic at 0 h PF. Scale bars represent 100µm. PF: Puparium Formation.

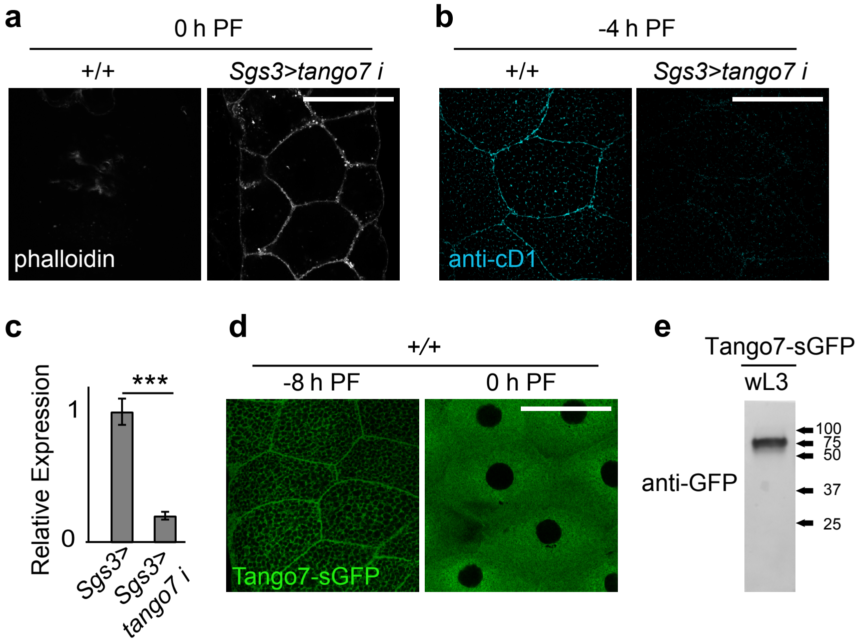

Supplementary Figure 5. *tango7* regulates caspase-dependent F-actin breakdown in salivary glands at the end of larval development. **(a)** Salivary-gland specific knockdown of *tango7* (using the *Sgs3-GAL4* driver) blocks F-actin dismantling, assayed by staining for phalloidin in white, at 0 h PF. **(b)** *tango7-RNAi* disrupts cortical anti-cD1 staining, in cyan, at -4 h PF. **(c)** qPCR analysis of *tango7* mRNA expression levels in control and *tango7-RNAi* salivary glands at 0 h PF confirms that *tango7* expression levels are significantly reduced upon expression of the RNAi. *y*-axis shows relative expression; *x*-axis shows the genotypes analyzed. Expression levels shown relative to control salivary glands and normalized to the reference gene *rp49*. Three biological samples analyzed for each stage; error bars represent standard error determined by REST analysis (see Methods); asterisks indicate *p*-value <0.05 calculated by REST analysis. **(d)** An endogenously-regulated, superfolder GFP-tagged Tango7 (Tango7-sGFP, in green) shows that Tango7 protein localizes to the salivary gland cell cortex at -8 h PF; this cortical localization is lost and Tango7-sGFP becomes cytoplasmic at 0 h PF. **(e)** Western blot analysis of Tango7-sGFP (using an anti-GFP antibody) shows a single band at the predicted molecular weight. Scale bars represent 100µm. PF: Puparium Formation.

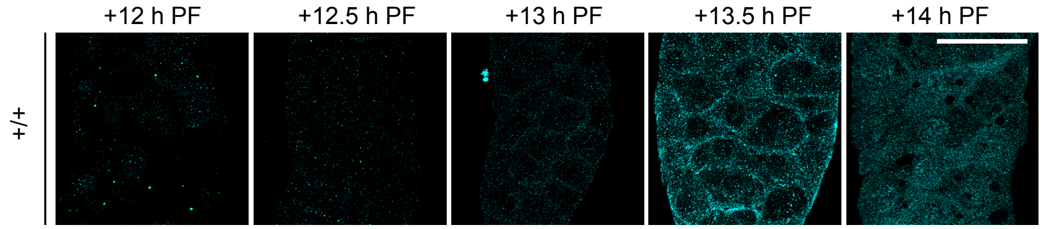

Supplementary Figure 6. Cortical anti-cD1 staining is a transient event during salivary gland cell death. Timecourse showing staining for anti-cD1, in cyan, in salivary glands every 30 min from +12 h PF to +14 h PF. No cortical or cytoplasmic staining is observed in +12, +12.5, or +13 h PF glands; however, +13.5 h PF glands exhibit robust cytoplasmic and cortical anti-cD1 staining. By +14 h PF, however, only cytoplasmic anti-cD1 staining is visible. PF: Puparium Formation.

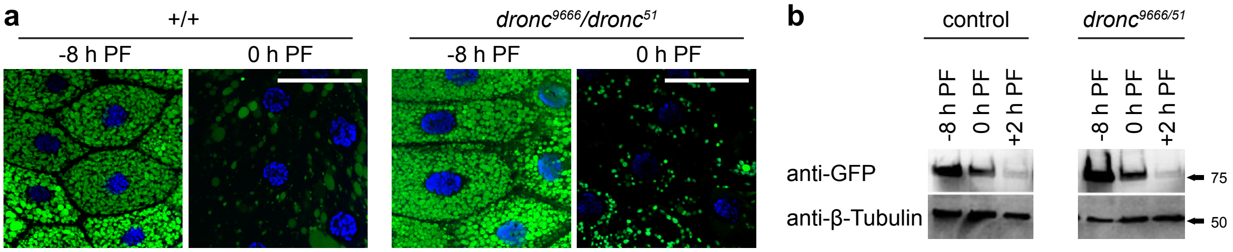

Supplementary Figure 7. *dronc* mutant salivary glands do not disrupt glue protein synthesis or secretion. **(a)** Live imaging of glue protein granules (visualized with Sgs3-GFP) before and after secretion in salivary glands. At -8 h PF, synthesized glue proteins are sequestered within secretory vesicles. At 0 h PF, when glue proteins are expelled onto the surface of the animal, there is little glue protein remaining inside salivary gland cells. *dronc* mutant glands are indistinguishable from control glands. **(b)** Assay of glue secretion using whole animal western blot analysis. Whole animals of appropriate stages were rinsed and analyzed for remaining glue proteins (western blots probed by anti-GFP and anti-Tubulin antibodies). In control animals, expulsion of glue proteins is completed by +2 h PF. Once again, *dronc* mutant glands are indistinguishable from control glands. All animals carry the *Sgs3-GFP* transgene; DNA stained with DAPI in blue. Scale bars represent 100µm. PF: Puparium Formation.

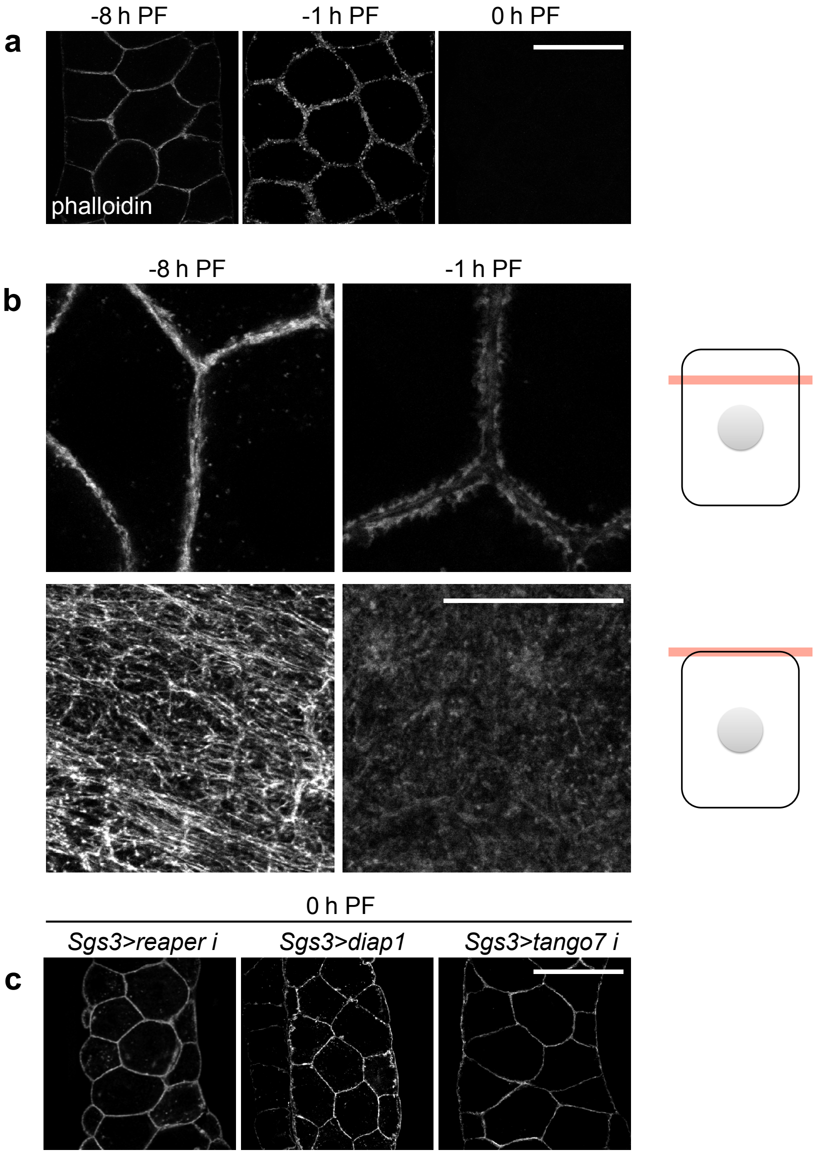

Supplementary Figure 8. Detailed analysis of F-actin breakdown in *rbp⁵* mutant salivary glands without luminal expansion. *rbp⁵*, a mutant of the ecdysone primary response gene *BR-C*, synthesizes and secretes very little glue protein; therefore, the lumen does not dramatically expand, enabling high-magnification analysis of F-actin breakdown. **(a)** Analysis of F-actin breakdown in *rbp⁵* mutant salivary glands. At -8 h PF, phalloidin staining, in white, shows F-actin in tight cortical bundles. By -1 h PF, the F-actin structure has begun to "fray," and by 0 h PF, F-actin is completely broken down. **(b)** High magnification analysis of F-actin structure in *rbp⁵* mutant salivary glands. At -8 h PF, a coronal confocal slice shows F-actin tightly bundled at the cortex, while a slice at the surface of the basal membrane shows a clear F-actin meshwork on the surface of the cell. In contrast, at -1 h PF, F-actin has begun to "fray" and fall away from the cortex, and the meshwork has dissolved. Diagrams on right show focal plane being imaged within the cell; gray circle represents the nucleus. **(c)** F-actin breakdown is caspase-dependent in *rbp⁵* mutant salivary glands. F-actin dismantling is blocked at 0 h PF upon overexpression of *rpr-RNAi*, *diap1*, or *tango7-RNAi* using the *Sgs3-GAL4* driver in the *rbp⁵* mutant background. All images show phalloidin staining in white. Scale bars represent 100μm. PF: Puparium Formation.

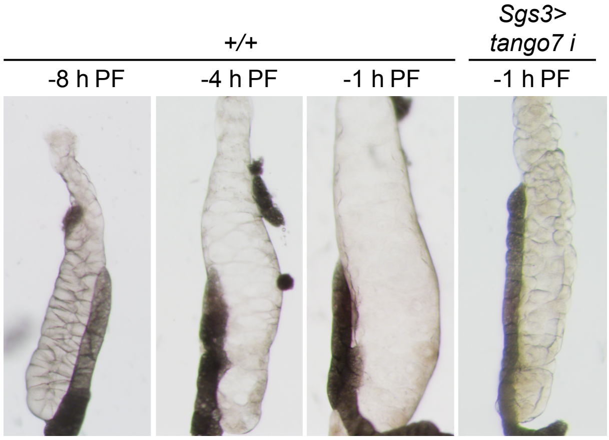

Supplementary Figure 9. Knockdown of *tango7* prevents salivary gland luminal expansion. Light microscope images of whole salivary glands during the end of larval development. Control glands are thin, with no luminal expansion, at -8 h PF. By -4 h PF, when glue exocytosis has begun, the lumen begins to expand, reaching maximal size at -1 h PF. In contrast, salivary glands expressing *tango7-RNAi* do not expand at -1 h PF. PF: Puparium Formation.

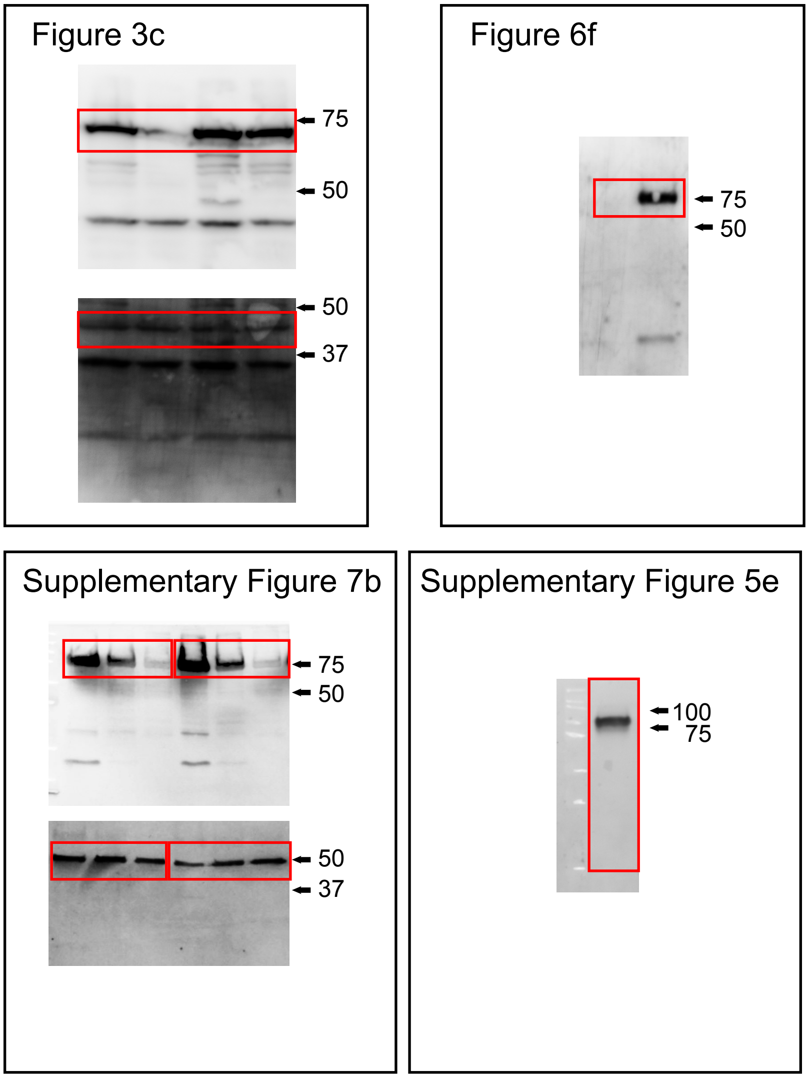

Supplementary Figure 10. Full-length western blots. For each blot, the red box indicates the portion that was cropped and displayed in the indicated figure.
